# Oxidative stress-induced premature senescence and aggravated denervated skeletal muscular atrophy by regulating progerin–p53 interaction

**DOI:** 10.1186/s13395-022-00302-y

**Published:** 2022-07-29

**Authors:** Yaoxian Xiang, Zongqi You, Xinying Huang, Junxi Dai, Junpeng Zhang, Shuqi Nie, Lei Xu, Junjian Jiang, Jianguang Xu

**Affiliations:** 1 Department of Hand Surgery, Huashan Hospital, Fudan University, Shanghai, People’s Republic of China; 2 NHC Key Laboratory of Hand Reconstruction, (Fudan University), Shanghai, People’s Republic of China; 3 Shanghai Key Laboratory of Peripheral Nerve and Microsurgery, Shanghai, People’s Republic of China; 4 Shanghai Medical College of Fudan University, Shanghai, People’s Republic of China; 5 School of Rehabilitation Science, Shanghai University of Traditional Chinese Medicine, Shanghai, People’s Republic of China

**Keywords:** ROS, NO, Denervated muscle atrophy, Progerin, P53

## Abstract

**Background:**

Progerin elevates atrophic gene expression and helps modify the nuclear membrane to cause severe muscle pathology, which is similar to muscle weakness in the elderly, to alter the development and function of the skeletal muscles. Stress-induced premature senescence (SIPS), a state of cell growth arrest owing to such stimuli as oxidation, can be caused by progerin. However, evidence for whether SIPS-induced progerin accumulation is connected to denervation-induced muscle atrophy is not sufficient.

**Methods:**

Flow cytometry and a reactive oxygen species (ROS) as well as inducible nitric oxide synthase (iNOS) inhibitors were used to assess the effect of oxidation on protein (p53), progerin, and nuclear progerin–p53 interaction in the denervated muscles of models of mice suffering from sciatic injury. Loss-of-function approach with the targeted deletion of p53 was used to assess connection among SIPS, denervated muscle atrophy, and fibrogenesis.

**Results:**

The augmentation of ROS and iNOS-derived NO in the denervated muscles of models of mice suffering from sciatic injury upregulates p53 and progerin. The abnormal accumulation of progerin in the nuclear membrane as well as the activation of nuclear progerin–p53 interaction triggered premature senescence in the denervated muscle cells of mice. The p53-dependent SIPS in denervated muscles contributes to their atrophy and fibrogenesis.

**Conclusion:**

Oxidative stress-triggered premature senescence via nuclear progerin–p53 interaction that promotes denervated skeletal muscular atrophy and fibrogenesis.

**Supplementary Information:**

The online version contains supplementary material available at 10.1186/s13395-022-00302-y.

## Background

Skeletal muscle atrophy progressively after losing innervation. This severely affects the functional reconstruction of the target muscle and burdens the patient and society with the cost of treatment [1, 2]. Although treatments to postpone muscle atrophy have had some success, the sluggish recovery of functions of the atrophic muscles remains a clinical problem [3, 4]. Elucidating the underlying pathological process after denervation may help us understand the pathogenesis of muscle atrophy. P53 is a crucial protein that participates in the pathological or physiological processes of various diseases. However, whether it has a unique effect on denervated skeletal muscle cells has not been adequately considered in research to date.

Past work has shown that a p53-dependent process may be crucial in repairing DNA-damaged cells after oxidative stress by controlling the cascade of irreversible malfunctions [5, 6]. However, if DNA damage is insufficient to cause cell death but the cell cannot be completely repaired, cell senescence may occur [7]. Premature senescence is a type of senescence characterized by p53-dependent irreversible G1 arrest. It can be caused by oncogene activation and various stresses [8, 9]. Reactive oxygen species (ROS) are a class of highly active stress oxygen molecules [10] that are considered to be an important determinant of genomic integrity and cell response to DNA damage [11]. A significant increase in the ROS level is a key event in inducing and maintaining cell senescence [12, 13]. The increase in mtDNA damage caused by various stresses may hinder electron transmission and lead to the excessive production of ROS, thus accelerating the premature senescence of cells [14]. In addition, low-dose H_2_O_2_ treatment significantly accelerates the process of telomere shortening, which may further trigger premature senescence through a DDR-ATM-p53-dependent pathway [15].

In the context of ROS-induced stress, iNOS-derived NO has been shown to be intimately connected with the modulation of muscle physiology [16], such as muscular dystrophy during the pathogenesis of aging-related diseases [17]. This pathology usually involves apoptosis mediated by nuclear factor p53 [18]. The effect of NO on apoptosis is mainly mediated by the S-nitrosylation of p53-related proteins [19]. Moreover, the increase in S-nitrosation may lead to cell damage and senescence. Research has confirmed that the excessive production of NO in the skeletal muscles can lead to oxidative damage and muscle atrophy [20]. Thus, oxidative stress may be important factor in the mediation of p53, cell senescence, and muscle atrophy. This motivates us to find the connections among them.

Aging-related changes to such biological structures as musculoskeletal systems contribute to the development of frailty and ultimately lead to premature death [21]. Some studies have used Slc25a46-/- mice to examine the phenotype of segmental premature senescence and have found that its main characteristics emphasize gastrocnemius atrophy [22]. Other studies have identified and used Tmem158 and CDKN1A as aging and denervation markers (SDMS), respectively, associated with muscle atrophy [8]. Past work has shown that the deletion of the ZMPSTE24 gene leads to an inability to form mature Lamin A from prelaminin A, which is accompanied by the production of progerin—a silent-point mutation in the Lamin A/C gene [23]—to result in the weakness and atrophy of the muscles of the lower limbs as well as reduced intrinsic strength of the muscle fibers and filaments [24]. The muscle-specific overexpression of progerin in mice significantly reduces muscle mass and myofiber size to halve grip strength [25]. Therefore, there is a close relationship between muscle atrophy and premature senescence, but whether a specific relationship obtains between denervated muscle atrophy and premature senescence is unclear.

In this study, we examine the expression of p53 and progerin and their interactions in the denervated muscles. We show that denervation induces an increase in ROS and iNOS-derived NO, which lead to the upregulation of progerin and p53. The skeletal muscles subsequently undergo premature senescence, eventually atrophy, and lead to fibrogenesis after denervation.

## Material and methods

### Experimental animals

Male P53 KO mice, aged 6 to 8 weeks and weighing 22–25 g each, were purchased from the Model Organisms Center (Shanghai, China). Male C57 BL/6 mice aged 6 to 8 weeks and weighing 22–25 g were purchased from the Vital River Laboratory Animal Technology Co. (Zhejiang, China). The mice were housed in standard cages in a room at 23°C and 50% relative humidity on a 12-h:12-h light/dark cycle. For qPCR, western and flow cytometry, four mice were used per each experimental group. For histology, six mice were used per experimental group. For ROS or INOS Inhibition, the mice received a sham operation were administered with saline (control group). For other experiments, the mice that received a sham operation were used as controls. For ROS inhibition, the denervated mice were administered with L-NAC (Den+anti-ROS group). For INOS inhibition, the denervated mice were administered 1400W (Den+anti-iNOS group). The mice in both denervated groups were anesthetized and subjected to unilateral sciatic nerve transection [26]. After deep anesthetization, a 0.5-cm-long portion of the sciatic nerve in the right hind leg of each mouse was resected; the two nerve ends were buried in the muscles, and the incision was closed using 4-0 absorbable sutures. The mice were randomly assigned to experimental groups for analysis at specific time points after denervation.

#### Wet weight

Seven and 14 days after denervation, the mice and control group were anesthetized, and the gastrocnemius muscles of both the left and the right hind legs were removed, washed with saline, and then weighed. The loss of muscle weight ratio was defined as the weight of the contralateral side minus the muscle weight of the side with the nerve injury divided by the weight of the contralateral side. The muscle samples were stored in 4% paraformaldehyde at −80°C until use.

#### Quantitative real-time PCR (qPCR)

The RNeasy kit (Qiagen, Valencia, CA, USA) was used to extract the total RNA from the gastrocnemius muscle. The cDNA was synthesized using a first-strand cDNA synthesis kit with oligo dT primers (Invitrogen, Carlsbad, CA, USA) and used for quantitative real-time PCR (qPCR) (MJ Research, Waltham, MA, USA). The thermal cycling conditions were as follows: 94°C for 5 min, 35 cycles at 94°C for 30 s, 55°C for 45 s, 72°C for 1 min, and 72°C for 5 min. The relative expression level of the target gene was calculated by using the cycle threshold (Ct) value. The expression levels of iNOS, α-SMA, progerin, and P53 were normalized to GAPDH levels. The primer sequences were as follows: iNOS: R, 5′ GACCTGATGTTGCCATTGT 3′, F, 5′ TTG ACG CTC GGA ACT GTA G 3′; α-SMA: R, 5′ CAC AGC CTG AAT AGC CAC ATA C 3′, F, 5′ CCT GAA GAG CAT CCG ACA CT 3′, progerin: R, 5′ CTC TCG CTG CTT CCC GTT AT 3′, F, 5′ TGG ATG CTG AGA ACA GGC TAC A 3′, P53:R, 5′ ACT CGG AGG GCT TCA CTT 3′, F, 5′ CAG GAG ACA TTT TCA GGC TTA T 3′.

### Western blot analysis

The frozen gastrocnemius muscle samples were homogenized in a radioimmunoprecipitation assay buffer containing 1 mM phenylmethylsulfonyl fluoride and the Protease Inhibitor Cocktail (Roche Applied Science). The lysates were centrifuged for 20 min at 12000 × g (4 °C), and the protein level in the supernatant was quantified with a bicinchoninic acid assay kit (Beyotime). The proteins were separated by SDS–PAGE (Beyotime) and transferred to a polyvinylidene difluoride membrane (Beyotime) that was blocked with 5% non-fat dry milk in tris-buffered saline at room temperature, followed by incubation with primary antibodies: mouse anti-progerin (1:200; Santa Cruz Biotechnology, Sc-81611, USA), rabbit anti-α-SMA antibodies (1:1000; Affinity Biosciences, AF1032, USA), rabbit anti-P53 (1:1000; Abcam, Ab32532, UK) and anti-acetyl-P53 (1:1000; Abcam, Ab183544, UK), and rabbit anti-iNOS antibodies (1:1000; Affinity Biosciences, AF0199, USA). After being washed three times, the membrane was incubated with the appropriate secondary antibody (Abcam) at room temperature for 1 h. The enhanced chemiluminescence detection reagent and an X-ray film were used to visualize the proteins.

### Immunohistochemistry

The expressions of the P53 and progerin in the gastrocnemius muscle were detected by immunohistochemistry. The sections were deparaffinized with xylene and rehydrated with ethanol, and antigen retrieval was performed in a 0.01-M citrate buffer (pH, 6.0) in a pressure cooker, followed by natural cooling to room temperature. The sections were incubated in 0.3% hydrogen peroxide at room temperature for 10 min; goat serum was used to block the sections for 15 min at room temperature, and they were then incubated overnight at 4°C with mouse anti-progerin (1:50; Santa Cruz Biotechnology, Sc-81611, USA) and rabbit anti-P53 (1:100; Abcam, Ab32532, UK). This was followed by treatment with horseradish peroxidase-conjugated goat anti-rabbit IgG antibody (ABclonal, 33301ES60, Wuhan, China) for 30 min at room temperature. Immunodetection was performed by using a diaminobenzidine solution according to the manufacturer’s instructions. After being washed, the sections were counterstained, dehydrated, and then coverslipped by using neutral gum sealant.

### Immunofluorescence

To identify the p53 and progerin proteins in the denervated muscles, double immunostaining was performed. The paraformaldehyde-fixed denervated muscles were incubated with the primary antibody followed by the secondary antibody and were subsequently mounted with DAPI. The primary antibodies included mouse anti-progerin (1:25; Cruz Biotechnology, Sc-81611, USA) and rabbit anti-P53 (1:200; Abcam, Ab32532, UK). The number of positive cells was observed by fluorescence microscopy (1 × 71, Olympus, Japan) and quantified by ImageJ software.

### Cell preparation and flow cytometry

#### Preparation of single-cell suspension of gastrocnemius muscle

The gastrocnemius muscle tissue was rinsed with phosphate-buffered saline (PBS), placed in a 6-cm Petri dish, and cut into small pieces (2–4 mm). DNAse I at 1 mg/mL was mixed with the 1640 medium, and 5 ml was added to the pieces of tissue. Tissue-block solution was added to a gentleMACS C tube, and 5 μl of type-II collagenase was added as well. The tissue block was then placed in the gentleMACS C tube with the enzyme solution, the tube was installed in the casing of the gentleMACS tissue processor, and the heating module was inserted. The 37C-mr-SMDK-1 program was used for dissociation; after the program had completed execution, the C tube was removed and briefly centrifuged to pellet the tissue debris. The resuspended cells were filtered with a 70-μm filter, and the resulting cell suspension was collected in 50-ml test tubes. The filters were rinsed with 10 ml of the RPMI 1640 medium, the rinse medium with resuspended cells was centrifuged for 5 min, and the supernatant was discarded. The resuspended cells were then counted and stained.

#### Preparation of peripheral blood monocyte suspension

The red blood cell lysate of mouse was added to 50 μl of an anti-coagulant. The mixture was diluted to 1 ml (BioLegend, Cat # 420301) and incubated at room temperature away from light for 15 min. The lysate was then centrifuged at 4°C at 350 rpm for 5 min, and the sample was observed for the presence of red blood cells. If the red blood cells were observed, 1 ml of red blood cell lysate at room temperature was added while avoiding light and centrifuged at 4°C for 5 min. Following this, 3 ml of PBS was added to the resuspended cells and centrifuged at 4°C for 5 min. Finally, the cells were resuspended and counted and stained.

#### Detection of ROS

For ROS detection, 3 ml of PBS was added to 1 ml of the single-cell suspension (3×10^6^ cells) to resuspend the cells. The cells were centrifuged at 4°C at 350 rpm for 5 min, and the supernatant was discarded. Following this, 1 ml of the serum-free medium was added to the cell pellet to dilute the 2',7'-dichlorodihydrofluorescein diacetate (DCFH-DA) solution 1:1000 so that the final concentration was 10 μmol/L. The sample was incubated at 37°C for 30 min through mixing by inverting the tube every 3–5 min so that the probe remained in contact with the cells. The cells were then centrifuged and washed with serum-free cell culture medium three times by centrifugation at 4°C at 350 rpm to fully remove the DCFH-DA that had not entered the cells. A total of 200 μl of serum-free medium was then added to resuspend the cells. The cells were then examined by flow cytometry and the data were analyzed using FlowJo software.

#### ROS inhibition

The acetylcysteine (L-NAC) (MCE, China) or solvent control group (saline) was injected intravenously at a dose of 1.1 mmol/kg/d [27] before denervation and injected continuously for 14 days after denervation.

#### INOS inhibition

The iNOS inhibitor 1400W (MCE, China) or solvent control group (saline) was injected intraperitoneally (200 μg/mouse) [28] 1 day before denervation and was injected continuously for 14 days after denervation.

#### Hematoxylin–eosin (HE) and Masson’s trichrome staining

Gastrocnemius muscle samples from the mice were fixed in 4% paraformaldehyde and embedded in paraffin. Five-μm-thick pieces of the samples were cut, and the sections were stained with hematoxylin–eosin (HE) (Beyotime, Shanghai, China) and Masson’s trichrome (Beyotime) to identify histopathologic changes. The mean area, diameter, and density of the myofibers were determined by blinded analysis using Image-Pro Plus 6.0 software (National Institutes of Health, Bethesda, MD, USA) from six randomly captured images per mouse under each set of experimental conditions.

#### Statistical analysis

All data are presented as mean ± SEM. Differences between groups were evaluated by using the two-sample *t* test, Mann–Whitney *U* test, or the *χ*^2^ test. One-way or two-way ANOVA was conducted and was followed by the Bonferroni post hoc test for comparisons among groups. Differences at different time points were evaluated by using the Friedman test. Statistical analyses were performed using SPSS v17.0 software (SPSS Inc., Chicago, IL, USA). *P* values < 0.05 were considered statistically significant.

## Results

### Elevated nuclear p53 and progerin are closely linked to cell premature senescence in denervated muscles

**Fig. 1:**
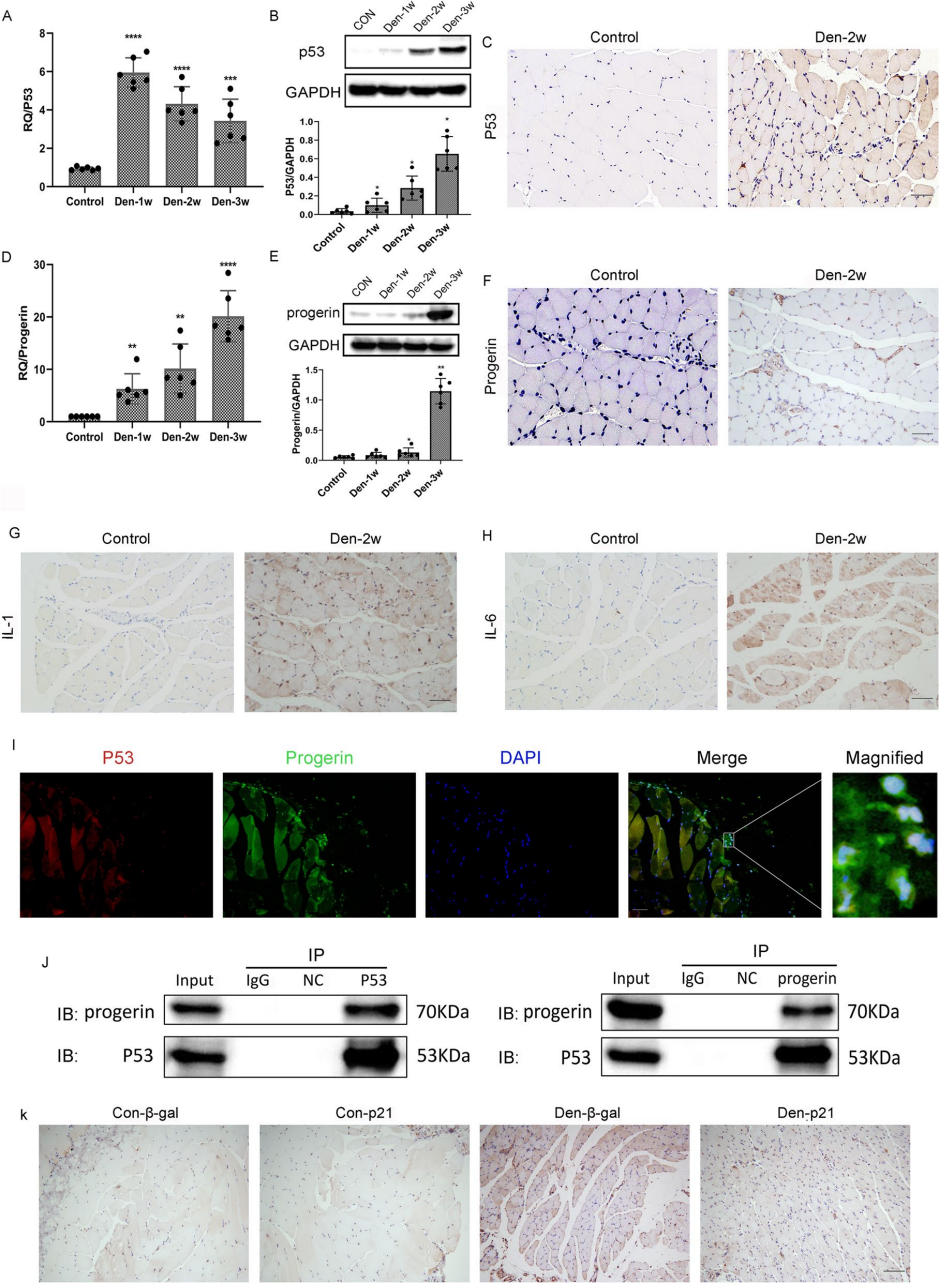
Elevated nuclear p53 and progerin are closely related to cell premature senescence in case of denervated muscle atrophy. **A** Elevated expression of the P53 mRNA in the WT group of mice after denervation was determined by qPCR. The level of elevation of P53 in mice in the Den group was much higher than that of mice in the control group. ***P<0.001 versus control, ****P<0.0001 versus control (*n*=6/group). **B** P53 protein levels determined by western blotting showed a trend similar to that of the mRNA expression. *P<0.05 versus control (*n*=6/group). **C** Compared with the muscles of mice in the control group, the positive expression of p53 increased in the denervated muscles of the Den-2w group of mice. **D** The elevated expression of progerin mRNA in the WT group after denervation was determined by qPCR. Elevation in progerin levels of mice in the Den-2w group was much higher than that of the control group. **P<0.01 versus control, ****P<0.0001 versus control (*n*=6/group). **E** Progerin protein levels determined by western blotting showed a trend similar to that of mRNA expression. *P<0.05 versus control, **P<0.01 versus control (*n*=6/group). **F** Compared with mice in the control group, the positive expression of progerin increased in the denervated muscle of mice in the Den-2w group. **G–H** Compared with muscles of mice in the control group, the positive expression of SASP-related factors such as IL-1 and IL-6 increased in the denervated muscles of the Den-2w group of mice. **I** The co-localization of p53 (red) with progerin (green) in the denervated muscle, shown by immunofluorescence. **J** Endogenous co-IP showed the positive interaction of p53 and progerin in denervated muscle. **K** ß-gal activity combined with p21 in sequential sections showed skeletal muscle cellular senescence. Scale bar, 50 μm

Compared with the control group, expressions of the p53 and progerin proteins, mRNA as well as staining in the group of mice with denervated muscle tissues were drastically higher (Fig. 1A–F). Intriguingly, p53 and progerin in the cell nuclei were highly expressed in the group with denervated muscle tissues (Fig. 1I, J). This suggests that the enhanced expression of nuclear p53 and progerin in the intramuscular cells may be substantially associated with premature cell senescence in the denervated muscle tissues of mice. Additionally, SASP-related factors such as IL-1 and IL-6 and ß-gal activity combined with concomitant marker p21 in sequential sections showed cellular senescence (Fig. 1G, H, and K).

### Premature senescence and P53 upregulation of muscle cells is initiated by ROS, with increase in iNOS-derived NO during denervation

**Fig. 2:**
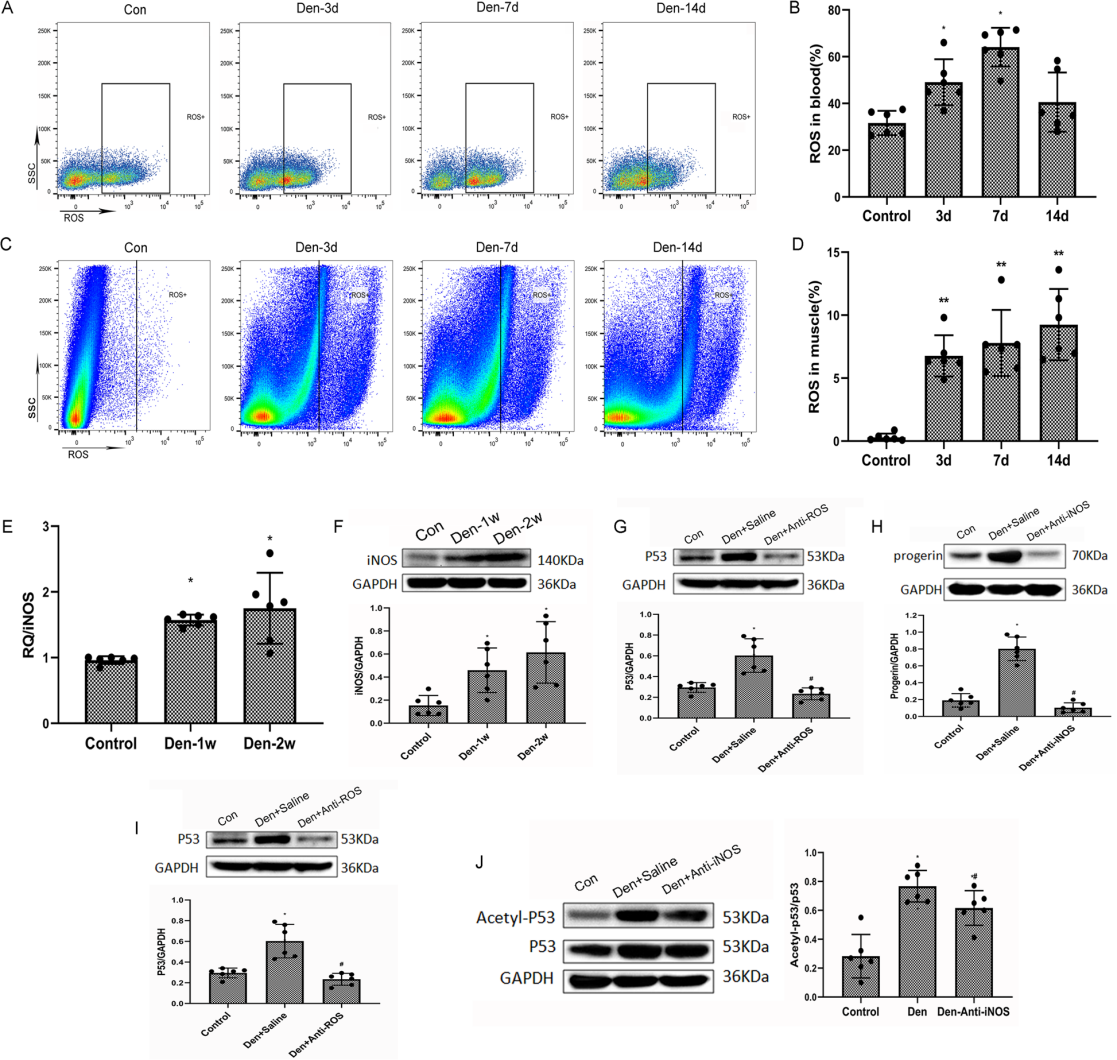
Increased ROS/iNOS-derived NO after denervation upregulated p53/progerin. **A** Flow cytometric analysis of ROS in the control WT peripheral blood group 3, 7, and 14 days after denervation. **B** Dynamic changes in ROS in the peripheral blood. *P<0.05 versus hour 0 (*n*=6/group). **C** Flow cytometric analysis of ROS in the control WT gastrocnemius muscles 3, 7, and 14 days after denervation. **D** Dynamic changes in ROS in denervated gastrocnemius muscles. *P<0.05 and **P<0.01 versus hour 0 (*n*=6/group). **E** Elevated expression of iNOS mRNA in the WT group of mice after denervation was determined by qPCR. Level of elevation of INOS of mice in the Den group was much higher than that of those in the control group. *P<0.05 versus control (*n*=6/group). **F** INOS protein levels determined by western blotting showed a trend similar to that of mRNA expression. *P<0.05 versus control (*n*=6/group). **G–H** The expression of progerin in the control, Den-saline, and the Den-anti-ROS/iNOS group of mice was determined by western blotting. Level of elevation of progerin in the Den-saline group of mice was much higher than that in the Den-anti-ROS/iNOS group. *P<0.05 versus control; ^#^*P*<0.05 versus Den-saline (*n*=6/group). **I–J** The expression of P53 in the WT group of mice after denervation was determined by western blotting. Level of elevation of P53 of the Den-saline group of mice was much higher than that of the Den-anti-ROS group. Levels of elevation of P53 and ac-p53 of the Den-saline group were much higher than those of the Den-anti-iNOS group. *P<0.05 versus control; ^#^*P*<0.05 versus Den-anti-saline (*n*=6/group)

We then performed the time-course flow cytometric analysis of ROS in both the peripheral blood and the denervated gastrocnemius muscles of the mice to characterize dynamic changes in ROS in vivo (durations of 3, 7, and 14 days; Fig. 2A, B). Compared with the control group, ROS expression in both the peripheral blood and the denervated gastrocnemius muscles were drastically higher (Fig. 2C, D). In addition, the iNOS mRNA and protein expressions in mice with denervated muscle tissues increased significantly compared with the control group (Fig. 2E, F). Elevated expressions of progerin in both groups of denervated mice were determined by western blotting. Progerin levels of the Den-anti-ROS/iNOS group of mice were much lower than those of the Den-saline group (Fig. 2G, H). The P53 level determined by western blotting showed a trend similar to that of progerin (Fig. 2I, J). These data suggest that oxidative stress activated the p53-dependent muscle senescence during denervation.

### Upregulated p53 after denervation contributes to denervated muscle atrophy

**Fig. 3:**
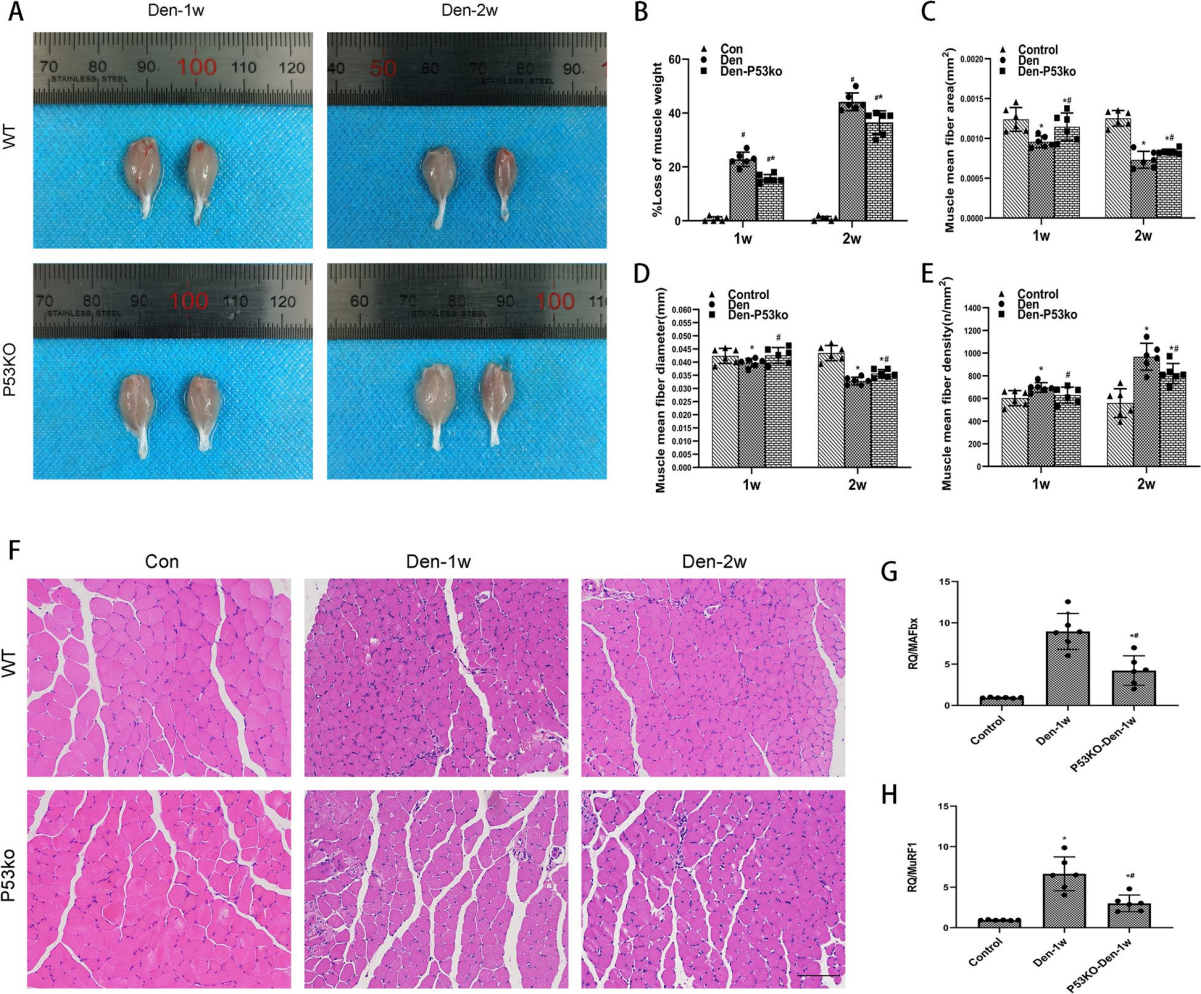
Attenuated denervated muscle atrophy using P53 KO. **A**, **B** Appearance and loss of gastrocnemius muscle weight of WT mice control, the P53 KO and WT mice at 1 and 2 weeks post-denervation. *P<0.05 versus Den (*n*=6/group). **C**, **D**, **E**, **F** HE staining of the muscle tissue, mean ± SEM fiber area, mean fiber diameter, and mean fiber density showing muscle atrophy that was ameliorated by p53 KO. *P<0.05 versus control; ^#^*P*<0.05 versus Den (*n*=6/group). Scale bar, 50 μm. **G**–**H** The expression of MuRF1 and MAFbx mRNA in the P53 KO and WT mice at 1 week post-denervation was determined by qPCR, showing muscle atrophy was ameliorated by p53 KO. *P<0.05 versus control; ^#^*P*<0.05 versus Den (*n*=6/group) WT, wild type; KO, knockout

To gain insights into the function of elevated p53 in denervated muscle atrophy, the loss-of-function approach was used in the model of denervated mice. The targeted deletion of P53 in the P53 KO mice resulted in a loss in p53. After 1 and 2 weeks of inducing sciatic injury in the P53 KO mice group, the denervation-induced loss of muscle weight was significantly alleviated (Fig. 3A, B), the mean fiber area (Fig. 3C), and the mean fibro-diameter of the gastrocnemius muscles (Fig. 3D) were larger, and their mean fiber density (Fig. 3E) was lower than in the WT mice. Atrophy-related markers such as MurF1 and Mafbx levels in mice with denervated muscle tissues increased significantly compared with the control group. The MurF1 and Mafbx levels of the P53ko-Den-1w group of mice were much lower than those of the Den-1w (Fig. 3G, H). Our results suggest that increased p53 can aggravate denervated muscle atrophy.

### Role of P53 in aggravating fibrosis in model of denervated muscle atrophy

**Fig. 4:**
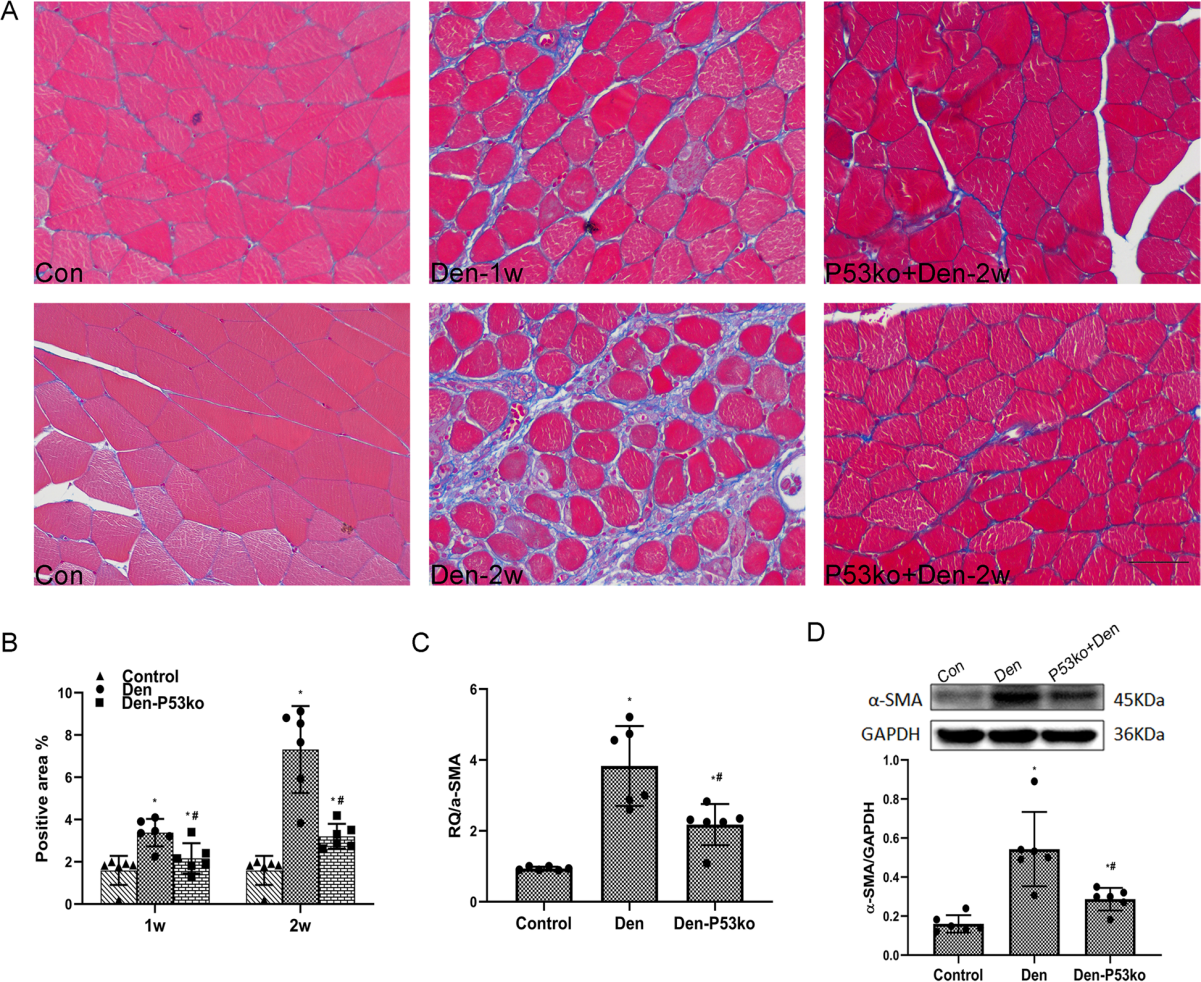
P53 KO attenuated muscle fibrosis after d enervation. **A**, **B** Masson’s trichrome staining of the muscle tissue and quantification of the positive area as percentages of the fiber area (blue collagen fiber), showing that muscle fibrosis was reduced when p53 was knocked out. Scale bar, 50 μm. *P<0.05 versus control; ^#^*P*<0.05 versus Den (*n*=6/group). **C** Elevated expression of α-SMA mRNA in both the WT and the P53 KO groups of mice after denervation was determined by qPCR. Level of elevation of α-SMA in the WT group of mice was much higher than the P53 KO group after denervation. *P<0.05 versus control; ^#^*P*<0.05 versus Den (*n*=6/group). **D** Levels of α-SMA protein determined by western blotting showed a trend similar to that of the mRNA expression. *P<0.05 versus control; #*P*<0.05 versus Den (*n*=6/group). WT, wild type; KO, knockout

We then assessed whether elevated p53-induced fibrosis in the denervated muscles by using a loss-of-function approach on the model of denervated mice. The targeted deletion of P53 in the P53 KO mice resulted in a loss of it. After 1 and 2 weeks of inducing sciatic injury in the P53 KO group of mice, the area density of Masson staining in their denervated muscle atrophy tissues were drastically lower than those of WT mice (Fig. 4A, B). Moreover, the elevated expression of fibrotic index α-SMA in both denervated groups of mice were determined by qPCR. The α-SMA mRNA expression of the Den-p53 KO group of mice was much lower than the Den group (Fig. 4C). The α-SMA protein level determined by western blotting showed a trend similar to that of mRNA (Fig. 4D). Our results suggest that upregulated p53 can worsen muscle fibrogenesis after denervation.

## Discussion

We have shown that the interaction between nuclear p53 and progerin aggravated the oxidative premature senescence of the skeletal muscles after denervation and eventually led to muscle atrophy and fibrosis. The principal findings are as follows: (1) The premature senescence of denervated muscle was initiated by oxidative stress. (2) ROS and iNOS-derived NO accelerated the premature senescence of the denervated muscles by activating p53/progerin interaction, resulting in muscle atrophy.

Stress-induced premature senescence (SIPS) is a special kind of senescence that plays a key role in regulating cell metabolism and function. It is usually caused by oxidative stress to prevent cell growth [29, 30]. Premature senescence caused by excessive stress can trigger the abnormal accumulation of senile cells, thus affecting cell function and tissue repair and leading to aging-related disorders and diseases [31]. Previous studies have shown that denervation also causes the target muscle tissue to produce excessive ROS [32, 33], which leads to the expression of downstream atrophy-related genes [34, 35]. We also discovered that ROS production increased significantly in the denervated muscle, as did ROS production in the circulation. In addition, oxidative stress often accompanies nitrosative stress [36, 37], and can enhance NO expression [38, 39]. In this study, we observed a significant increase in the expression of iNOS-derived NO. Furthermore, other studies have found that EXOSC2 mutations can induce premature senescence [40] and nucleotide pool imbalance, leading to neuronal defects. These in turn explain such related diseases as amyotrophic lateral sclerosis and spinal muscular atrophy [41]. Hence, it is important to examine whether oxidative premature senescence runs through the progression of denervated muscle atrophy.

We also explore the mechanisms of stress-induced muscle denervation. Prelamin A and, subsequently, the mature Lamin A/C protein constitute the nuclear lamina and the nuclear matrix, and are involved in a variety of nuclear activities to maintain cellular stability and activity [42, 43]. Nevertheless, mutations of prelamin A and Lamin A/C trigger multiple tissue disorders and a series of diseases. Progerin is a kind of mutant prelamin A protein that is a special marker of cell premature senescence. Moreover, the abnormal accumulation and distribution of progerin is a hotspot for association with cell dysfunction and a variety of diseases [44, 45]. Emerging reports have shown that lamins play a critical role in the nucleoskeleton and the cytoskeleton, whereas the accumulation of progerin contributes to perturbations in actin organization, implying that progerin-associated premature senescence modulates the cytoskeleton to affect cell phenotype [46, 47]. We found that the expression of progerin was significantly upregulated in the gastrocnemius muscles of the mice after denervation. We also carried out ROS or the iNOS block test and found that the levels of progerin had been downregulated. Hence, we showed that muscle premature senescence activated by oxidative stress emerged in muscles in the denervated mouse models.

Previous studies have shown that oxidative stress can aggravate progerin-associated premature senescence via the acetylation of p53 in the liver [47]. Other studies have reported that lamin-related ageing is regulated by transcription-related factors of p53 [48]. In addition, the upregulated expression of p53 and p21 induced by chronic hydroxyurea can lead to the premature senescence of cells [49]. In previous studies, we showed that the p53 signaling pathway is among the 20 most enriched KEGG pathways according to the high-throughput transcriptome sequencing of denervated muscle samples of human. In this study, we found that both mRNA and the protein levels of p53 were upregulated in the denervated muscles of mice. We used the ROS or iNOS inhibitor after denervation and found that the expression of P53 had sharply decreased. In particular, the proportion of ac-p53 in the denervated muscles had decreased significantly after the use of the iNOS inhibitor. Progerin is the same as several other pathological changes associated with nuclear membrane protein gene defects, including Emery-Dreifuss muscular dystrophy, limb-Girdle muscular dystrophy, and congenital muscular dystrophy, which can lead to severe muscle pathology similar to muscle weakness in the elderly, suggesting that modification on the nuclear membrane may alter the development and function of skeletal muscle cells [50]. Interestingly, we found that more progerin was co-localized with p53 in the nuclear envelope of the muscle cells after denervation. These results indicate that oxidative stress triggered premature senescence associated with the interaction between progerin and p53.

Mutations in the LMNA gene disrupt the expression and function of Lamin A in the nuclei of all cell types and can lead to altered forms known as progerin [23, 51]. SUN1 accumulation in LMNA mutant mouse fibroblasts and human HGPS patient fibroblasts is known to be associated with nuclear defects and cellular senescence [52]. Altered SUN1 expression or distribution in the skeletal muscles can impair mechanotransduction by elevating atrophic gene expression and suppressing the expression of genes encoding contractile proteins [50]. Given that p53 activation is linked to the loss of function of the progenitor cells and induction of cellular senescence [53] as well as the co-localization of p53 and progerin in the nuclear envelope of the muscle cells after denervation, we used the denervated muscles of P53 KO mice to show that denervated muscle atrophy can be attenuated after P53 KO. We demonstrated that the co-expression of p53 and progerin contributes to denervated skeletal muscle atrophy.

Previous studies have shown that p53 is drastically upregulated within the AT2 cells of the explant tissue in idiopathic pulmonary fibrosis (IPF) [54]. Other studies have shown that senescence, rather than the loss of alveolar type 2 (AT2) cells, promotes progressive fibrosis, and that either genetic or pharmacologic interventions targeting p53 activation or senescence can block fibrogenesis. P53-induced AT2 senescence serves as a proximal driver and therapeutic target in progressive lung fibrosis [55, 56]. Thus, these data identify TP53/Trp53 as a critical regulator of fibrosis in epithelial samples of both humans and mice. In this study, we found that denervated muscle atrophy and fibrogenesis can be attenuated after P53 KO. These data indicate that dysfunctional and prematurely senescent denervated muscle cells, induced by oxidative stress, likely initiate atrophy and fibrogenesis via p53–progerin interaction.

## Conclusions

**Fig. 5:**
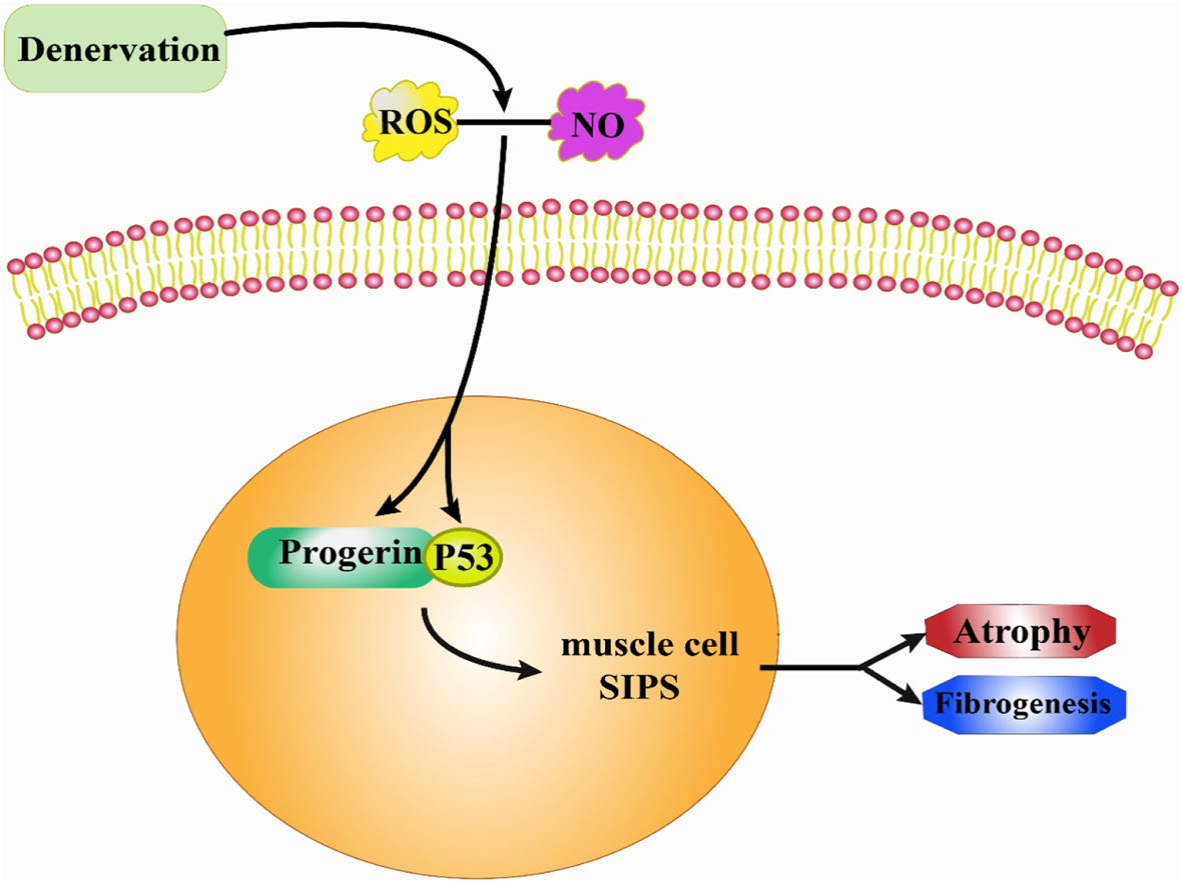
Proposed model of major pathway of signal transduction. ROS- or NO-aggravated denervated skeletal muscular atrophy and fibrogenesis by regulating nuclear progerin–p53 interaction

Collectively, the interaction between p53 and progerin in the nuclei increase the premature senescence of denervated muscle cells, which promotes progressive denervated muscle atrophy and fibrosis based on p53 (Fig. 5). Either genetic or pharmacological interventions targeting p53 activation or premature senescence can block denervated muscle atrophy and fibrogenesis.

## Supplementary Information

**Additional file 1.**

## Data Availability

All data generated or analyzed during this study are included in this published article and its supplementary information files.
