# Supplementary material for: Oxidative stress-induced premature senescence and aggravated denervated skeletal muscular atrophy by regulating progerin–p53 interaction
Source: Skelet Muscle. 2022 Jul 29;12:19. doi: 10.1186/s13395-022-00302-y (PMC9335985; doi:10.1186/s13395-022-00302-y)
Supplement: Supplementary file 1 — Additional file 1. [file 13395_2022_302_MOESM1_ESM.docx]

1. The number of ßgal/p21 of positive cell showed the average effect across the full n=6 used for such purpose.


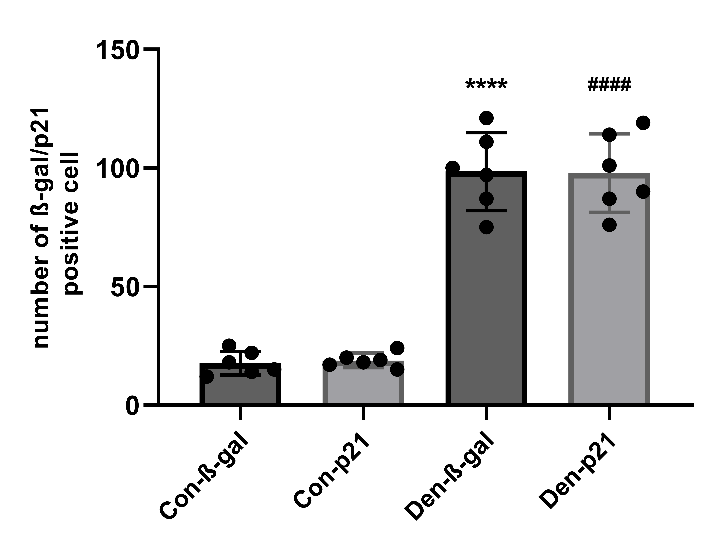


1.
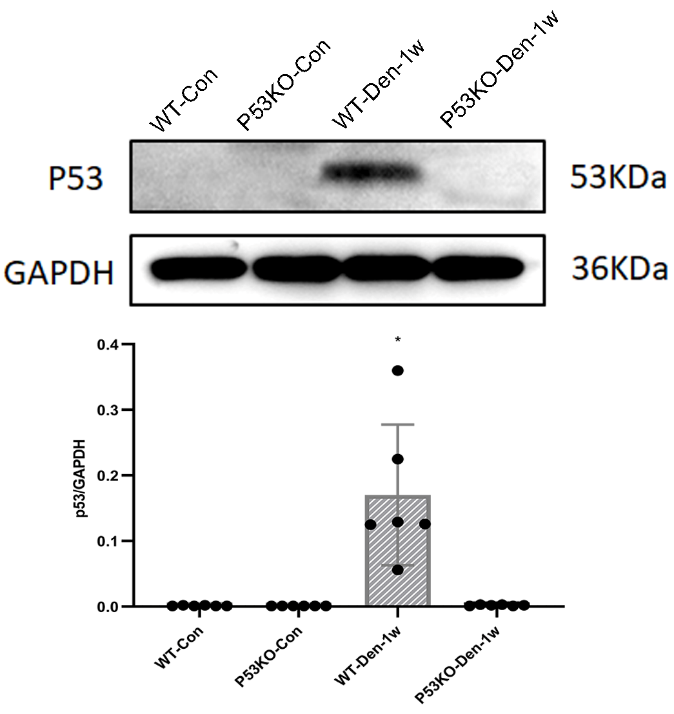
We added the western date to on the WT mice to compare to the KO.

3. We presented GAPDH expression levels to show that the expression of the "housekeeping" gene was consistent.
